# A review of UK publicly funded non-inferiority trials: is the design more inferior than it should be?

**DOI:** 10.1186/s13063-024-08651-3

**Published:** 2024-12-04

**Authors:** Nikki Totton, Steven Julious, Stephen Walters, Elizabeth Coates

**Affiliations:** 1 Sheffield Centre for Health and Related Research (SCHARR), University of Sheffield, Regent Court, 30 Regent Street, Sheffield, S1 4DA UK; 2 Department of Neuroscience, University of Sheffield, Sheffield, UK

**Keywords:** Non-inferiority trials, Non-inferiority margin, Margin justification, Analysis population, Review

## Abstract

**Background:**

The number of non-inferiority (NI) trials, those aiming to show a new treatment is no worse than a comparator, is increasing. However, their added complexity over superiority trials can create confusion. Most guidance and reviews to date have an industry focus with research suggesting these trials may differ from publicly funded NI trials. The aim of this work is to review the design and reporting characteristics of UK publicly funded NI trials. This assessment will show how well recommendations from industry are translating to publicly funded trials.

**Methods:**

The International Standard Randomised Controlled Trial Number web registry and the National Institute for Health and Care Research’s Funding and Awards Library and Journals Library were searched using the term non-inferiority and logical synonyms. Inclusion requirements were a UK publicly funded NI randomised controlled trial. Characteristics of the design, analyses and results as available were recorded on a dedicated data extraction spreadsheet. Appropriate summary statistics were used to present the results.

**Results:**

Searches completed on the 14th of January 2022 identified 477 potential trials which after exclusions resulted in a database of 114 NI trials to be summarised. Non-inferiority margins were defined for most trials with a median of 8% (IQR: 3–10%) used for risk differences (*n* = 58) and 0.35 (IQR: 0.26–0.43) standardised mean difference for continuous outcomes (*n* = 30). Justifications for the margin chosen (*n* = 62) were more commonly based on the clinical importance (49/62) and less commonly using statistical considerations (13/62). The most prevalent primary analysis population was solely on an intention-to-treat basis (49/114). The superiority of the treatment was well described but not always included as an outcome and only powered for in about a third of cases.

**Conclusions:**

Aspects of NI trial design are well described but not always in line with current recommendations. Of particular note, is the absence of statistical considerations when setting the non-inferiority margin, which eliminates the ability to confirm indirect superiority over placebo for the new treatment. Additionally, despite suggestions that it can increase the type 1 error in NI trials, the use of the intention-to-treat alone is the most common analysis population.

**Trial registration:**

Research on Research ID: 3171 (registration date: 31st May 2023).

## Background

Non-inferiority (NI) trials are used to test whether a new treatment is ‘no worse than’ a comparator [1]. This comparator is usually an existing treatment already in current practice, making the use of a placebo group unethical [2]. When considering UK publicly funded health care services, over time, more treatments are being implemented into the National Health Service (NHS). Subsequently, the popularity of publicly funded NI trials is increasing [3] to test against these treatments already used in practice. These trials become worthwhile to complete due to the added benefit that the new treatment has over that used in current practice. This benefit is related to an aspect other than the main health outcome which is being used as the primary outcome within the trial. This benefit may relate to safety, cost or convenience [4].

Many researchers have described the additional complexity that designing NI trials brings compared with the more common superiority trials, one element of which being the choice of non-inferiority margin (NIM) [5–8]. The NIM is used to define the maximum acceptable difference between the treatments that would be considered non-inferior [9]. Therefore, the value chosen for the NIM is crucial in defining the success of the trial.

There are guidelines available [2, 10–13] which advise on selecting the most appropriate NIM. A recent survey, however, found that these guidelines were followed by only 44% of respondents suggesting uptake of this advice in practice is not high [14]. Additionally, previous reviews of NI trials suggest that although the NIM used was well reported, it was not well justified with under half of the NI trials providing this justification [15, 16]. By not justifying a margin it reduces the ability for readers to assess its appropriateness.

A summary of the guidelines and their recommendations can be found in Table 1 which consistently suggests that NIMs should be based on both statistical and clinical considerations. Statistical considerations reflect the need for the new treatment to be superior to placebo if an indirect comparison was to be made [2]. Practically, this requires the use of historical data from placebo-controlled trials of the comparator which will be used within the NI trial [10]. Clinical considerations confirm that the difference would be clinically irrelevant in practice [12]. Conflicting results have come from previous reviews which evaluated the existence of both statistical and clinical considerations, with some suggesting statistical considerations are more prevalent [16–19] and others favouring clinical considerations [15, 20–24]. These differences may be due to the inclusion criteria used within each of the reviews.

**Table 1:** Summary of guidelines and recommendations for non-inferiority trials

Guidance Document	Setting the non-inferiority margin (NIM)	Analysis populations	Significance level and power
**International Conference on Harmonisation (ICH)** [10, 11]	Focus on clinical justification of the NIM as well as historical data on the active control against placebo (i.e., statistical considerations)	Intention-to-treat analysis may bias towards showing non-inferiority	A one-sided test recommended to be used but the significance level considered separate to this decision
**European Medicines Agency (EMA)** [2, 42]	NIM should be “justified on both clinical and statistical grounds”. No specific method is recommended. The feasibility of the sample size should not be used as a reason for selecting a larger NIM	The ITT analysis set as well as the PP analysis set have equal importance in NI trials and therefore should be consistent to allow for “robust interpretation”	States that a two-sided 95% (equivalent to a one-sided 97.5%) confidence interval is used for the analysis
**CONSORT extension for NI trials** [12]	NIM should be considered both clinically and statistically. Statistical methods such as fixed-margin or synthesis methods are recommended for the statistical considerations	Non-ITT analyses can protect against increased risk of bias. Confidence in the results is best when both ITT and non-ITT results are consistent	States they must be defined but does not specify values
**Food and Drug Administration (FDA)** [13]	Recommend a two-margin approach, where M1 is defined using statistical considerations to be superior to placebo. M2 is a proportion of M1 defined based on clinical considerations as, “the largest loss of effect that would be clinically acceptable”[13]. M2 should always be smaller than M1. The use of the fixed-margin or the synthesis method is recommended	ITT is biased towards the null hypothesis of non-inferiority which causes problems for the trial validity. Imputation of missing data could counter bias due to attrition	Two-sided 95% confidence interval typically used to match statistical test of a superiority trial. Mention of using a stricter significance level but not a less stringent one Adequate statistical power required but no specification of the value

There are further complexities when designing and analysing NI trials such as the choice of analysis populations, selection of significance level (alpha) and the power (1-beta) used in the sample size calculation. Recommendations from the guidelines for each of these are also summarised in Table 1. When highlighting these issues, Pocock [25] provides opinions which align with the guidelines suggesting that both intention-to-treat (ITT) and per protocol (PP) analysis populations should be used and be consistent to confirm NI and a one-sided 2.5% significance level should be used as this is equivalent to the two-sided 5% level used in superiority trials.

Guidelines (as shown in Table 1) are predominantly authored by regulatory bodies therefore providing a focus on industry trials and so how these translate to publicly funded trials is not clear. Subsequently, most of the previous reviews have a heavy focus on industry-funded trials so again how publicly funded NI trials are being designed is not well represented in the literature. Two reviews found key differences when comparing NI trials from different funding sources [26], particularly around the methods used to set the NIM [19] so it is important to assess this in practice. Additionally, the added benefit may be different for publicly funded trials, where more broad benefits such as costs to the NHS or benefits to public health may be important considerations.

## Methods

### Aims

The aim of this review is to assess the design features of publicly funded non-inferiority trials. The specific objectives are to assess:

- Characteristics of the NIM; including the outcome it is based on and how it has been justified,
- Analysis population used/to be used for the primary analysis,
- Significance level (alpha) and power (1-beta) used in the sample size calculation,
- Characteristics of the added benefit; including the superiority outcome/s used to represent this, whether they have been considered as co-primary outcomes, who they are aimed to benefit (i.e. patient or NHS) and whether this benefit was considered in the justification for the NIM.

### Data identification and screening

Publicly funded NI trials were identified through the International Standard Randomised Controlled Trial Number (ISRCTN) web registry [27]. Additionally, as the largest funder within the UK for health and social care research [28], the National Institute for Health and Care Research’s (NIHR) Funding and Awards Library [29] and Journals Library [30] were also used.

The search terms for all databases were “non inferior”, “non-inferior”, “non inferiority” and “non-inferiority”. No date ranges were specified so available dates were based on database limits (NIHR Awards Library = July 1995, NIHR Journals library = 1997 and ISRCTN = 2000).

Inclusion criteria were as follows:

- Randomised controlled trial including a non-inferiority comparison,
- Publicly funded—defined as Government or public organisation [31],
- UK funder.

Exclusion criteria were being a pilot or feasibility study (as explicitly defined by the trial team) as NIMs may not have been defined at this point.

### Data extraction

Identified NI trials had details extracted from any available sources, including those contained within the databases, as well as available published or linked documentation such as protocols or results articles. The extraction spreadsheet was trialled with the first five NI trials to assess appropriateness [32] before being finalised. The extraction spreadsheet included the following sections (full list of extracted information in Appendix):

1. Administration (ID numbers etc.),
2. Trial characteristics including funding information,
3. Non-inferiority margin characteristics,
4. Statistical properties of the trial, and
5. Added benefit characteristics.

Selection and extraction were completed by NT and a 10% sample was checked by a second independent researcher.

### Analysis

Findings from the review are presented using basic summary statistics, for example, frequencies and percentages for categorical variables and means, standard deviations or medians and inter-quartile ranges (IQR) as appropriate for continuous variables.

The values for the NIM have been reported as described by the trial team. Additionally, for absolute risk differences, the relative differences were also calculated by dividing the difference by the control group event rate where available. The standardised mean difference was calculated for any continuous outcomes with both the mean and standard deviation reported. The hazard ratio was calculated so values were above one (regardless of the direction of the outcome) allowing a meaningful summary measure of the size of the effect.

The PRISMA statement for systematic reviews [33] has been followed where applicable for reporting the results of this study and the checklist and flowchart completed.

## Results

### Screening

All databases were searched by NT on the 14th of January 2022. The ISRCTN webpage identified 406 trials, the NIHR Funding and Awards Library identified 113 trials with the NIHR Journals Library finding an additional ten. After the removal of duplicates, there were 477 records to be screened.

**Fig. 1:**
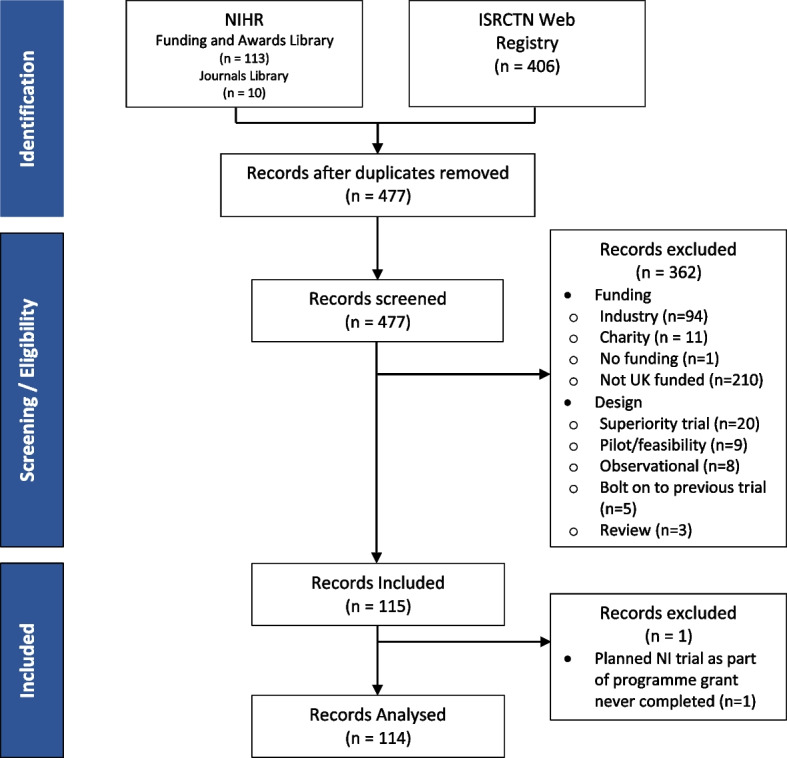
Flowchart for the database search and screening process

At screening, 362 trials were excluded with the two most common reasons being non-UK (*n* = 210) or industry (*n* = 94) funded. Five of the trials were excluded as they were bolt-on studies to an original NI trial, for each of these it was checked the original trial had been included. During extraction, one further entry was removed as it was a programme grant which intended to include a pilot NI trial but did not progress to this stage. After exclusions, 114 NI trials were left to have their information extracted. Full screening numbers can be found in the flowchart in Fig. 1.

### NI trial characteristics

The characteristics of the trials included in the review are shown in Table 2. On average, the NI trials had a target sample size of 720 (IQR: 393–1204) participants although this ranged greatly with some large, population-based trials (max = 360,000 participants). Such sample sizes have resulted in expensive trials, with the median trial costing £1.77 million (IQR: 1.34–2.32) and on average, lasting 5 years (IQR: 4.0–6.8). As would be expected, the vast majority (101/114, 89%) were funded by the NIHR and just over half of these (58/101, 57%) were through a researcher-led call, i.e. no specific topic or suggestions were provided by the funder for the focus of the research. The included NI trials were at a range of stages, with most being either in the recruitment phase (39/114, 34%) or having already been completed (41/114, 36%).

**Table 2:** General characteristics of the NI trials included in the review (*n* = 114)

**Characteristic**	**Median (IQR)**	**Range**
Trial duration (*N* = 114)	5.0 years (4.0–6.8 years)	0.5–16.0 years
Funding amount (*N* = 101)	£1,772,044 (£1,343,394–2,319,114)	£122,051–£13,984,021
Target sample size (*N* = 111)	720 (393–1204)	40–360,000
Number of treatment groups (*N* = 114)	2 (2–2)	2–5
**Characteristic**		**Frequency (%)** *N* = 114
Funder	National Institute for Health Research (NIHR)	101 (89%)
	*Researcher Led*	*58 (57%)*
	*Commissioned Call*	*43 (43%)*
	National Health Service (NHS)	7 (6%)
	Other public funder	6 (5%)
Project status	Complete	41 (36%)
	Recruitment	39 (34%)
	Set-up	15 (13%)
	Follow Up	12 (11%)
	Write-Up	6 (5%)
	Paused	1 (< 1%)
Health category	Cancer	21 (18%)
	Infection	20 (18%)
	Mental Health	10 (9%)
	Respiratory	10 (9%)
	Other	53 (7%)
Treatment type^a^	Drug	86 (34%)
	Surgery	46 (18%)
	Decision making	24 (10%)
	Medical device	17 (7%)
	Other	77 (31%)

^a^Trials can contribute to more than one treatment type in total 250 different treatments being used, percentages out of 250

The most common health categories were cancer (21/114, 18%) and infection (20/114, 18%). For the cancer trials, many of the rationales for completing a NI trial were based around the intensity and toxicity of cancer treatments whereas the rationale for infection NI trials was often the issue of antibiotic resistance. Drug treatments were the most common (86/250 treatments used within the trials, 34%) followed by surgical interventions (46/250, 18%).

**Fig. 2:**
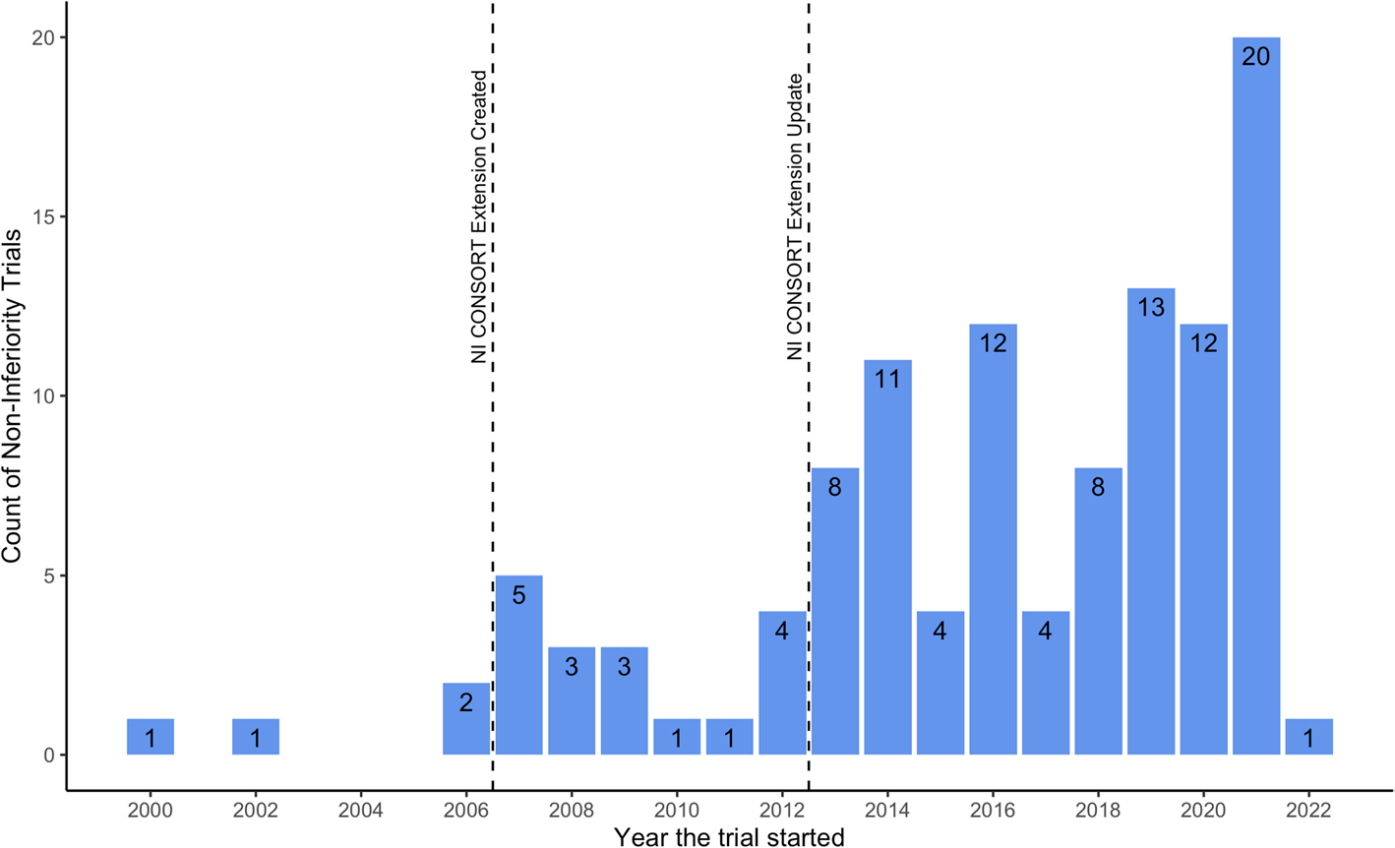
Starting year of NI trials included in the review (*n* = 114)

Figure 2 shows the increase in NI trials over time by presenting the year the NI trial started. The use of non-inferiority as a term is expected to increase after the publication of the NI CONSORT Extension [34] and its update [12] which recommended specific use of the term. Nevertheless, the large increase in numbers still suggests an increase in popularity.

### Non-inferiority margins

Most trials, 56% (64/114), had a binary primary NI outcome which has resulted in half (51%, 58/114) of the trials using a difference in absolute proportions, i.e. a risk difference, as the NIM (Table 3). The average absolute proportion difference used for the NIM (*n* = 58) was 8% (IQR: 3–10%), when calculated on a relative scale considering the control group rate, this represents a median of 20% (IQR: 9–39%) relative change. The standardised mean difference of continuous outcomes (*n* = 30) found a median value of 0.35 (IQR: 0.26–0.43), deemed to be a small to medium effect size [35].

**Table 3:** Values of the non-inferiority margin (NIM) and the assumed difference

**Non-inferiority margin**	**Frequency (%)** *N* = 114
**Difference in proportions (risk difference)**	58 (51%)
** Risk difference (*n* = 58)**	Median (IQR) = 0.08 (0.03–0.10) Min, max = 0.005, 0.37
** Calculated relative proportion difference (*n* = 53)**	Median (IQR) = 0.20 (0.09–0.39) Min, max = 0.17, 1.25
**Difference in means**	34 (30%)
** Calculated standardised mean difference (*n* = 30)**	Median (IQR) = 0.35 (0.26–0.43) Min, max = 0.13, 1.11
**Difference in median times**	4 (4%)
**Hazard ratio**	4 (4%)
** Hazard ratio (*n* = 7)**^a^	Median (IQR) = 1.33 (1.31, 1.38) Min, Max = 1.25, 1.43
**Odds ratio**	1 (1%)
** Odds ratio (*n* = 1)**	Median = 0.79 (0.79, 0.79)
**Unknown (not enough information)**	13 (11%)
**Assumed difference**	**Frequency (%)** *N* = 114
**Zero**	30 (26%)
**Not zero**	7 (6%)
** In favour of control** ** In favour of treatment**	5 (71%) 2 (29%)
** Not reported**	54 (47%)
** Unknown (not enough information)**	23 (20%)

^a^Hazard ratio calculated for difference in median time and included in summary

The assumed difference between the treatment groups that were used within the sample size calculation was only reported in 32% (37/114) of the NI trials. When it was reported, most of the cases (81%, 30/37) assumed a difference of zero. Of the remaining seven, five trials assumed the intervention to have a slightly worse outcome with only two of the trials assuming the intervention would be slightly better. This assumed difference was not reported in 47% (54/114) of cases, despite it being essential to the replication of the sample size calculation.

#### Justification

There was a justification presented for the chosen NIM for 54% (62/114) of trials within the review, however, 21% (24/114) of the trials did not provide any justification at all despite sufficient documentation being available where this information should be reported (Table 4).

**Table 4:** Justification basis for the value of the non-inferiority margin

Characteristic		Frequency (%)
**Has the NIM been justified? (** ***n*** ** = 114)**	Yes	62 (54%)
	No	24 (21%)
	Unknown (not enough information)	28 (25%)
**Justification used (** ***n*** ** = 62) **^a^	Clinically unimportant difference	49 (79%)
	*Clinician judgement*	*30 (26%)*
	*Patient judgement*	*20 (18%)*
	*Previously defined meaningful differences*	*13 (11%)*
	Statistical considerations	13 (21%)
	*Superior to placebo using control efficacy*	*10 (16%)*
	*Detectable difference*	*3 (5%)*
	Previous Non-Inferiority Trials	13 (21%)
	Guidelines	9 (15%)
	*Disease-specific guidelines*	*6 (10%)*
	*Guidelines for non-inferiority trials*	*3 (5%)*
	Other	2 (3%)

^a^Trials can contribute to more than one justification so percentage will not sum to 100%

Statistical considerations were present in 21% (13/62) of justifications with most of these, however not all, being related to confirming superiority over placebo. Clinical considerations, i.e., confirming the difference is clinically unimportant, were much more prevalent being mentioned in 79% (49/62) of the justifications. This was often through consulting clinicians (30/49) but it was also common to see patient input included (20/49). The recommendations for considering both statistical and clinical considerations have only been implemented in 6 of the 62 trials which have a justification (10%).

### Analysis populations, significance level and power

In terms of primary analysis populations (Table 5), 43% of trials (49/114) selected to use intention-to-treat (ITT) only, with the next most common being both ITT and Per Protocol (PP) (15%, 17/114). Per protocol definitions were not assessed for consistency between trials and were identified by author-declared PP analysis. There was a small proportion (6/114, 5%) that did not specify the analysis population that they were using.

**Table 5:** Statistical characteristics of the NI trials (*N* = 114) included in the review

**Characteristic**		**Frequency (%)** *N* = 114
Primary analysis population	Intention-to-treat only	49 (43%)
	Intention-to-treat and per protocol	17 (15%)
	Per protocol only	11 (10%)
	Other	7 (6%)
	Not reported	6 (5%)
	Unknown (not enough information)	24 (21%)
Power (1-beta)	90%	57 (50%)
	80%	27 (24%)
	Other	15 (13%)
	Not reported	1 (1%)
	Unknown (not enough information)	14 (12%)
Significance level (alpha)	0.025 one-sided/0.05 two-sided	61 (54%)
	0.05 one-sided	29 (25%)
	Other	5 (4%)
	Not reported	1 (1%)
	Unknown (not enough information)	18 (16%)

The significance level, summarised in Table 5, shows most NI trials and a 2.5% one-sided alpha (or equivalently 5% two-sided) within the sample size calculation (54%, 61/114), as recommended. However, it was not uncommon for a one-sided 5% alpha (25%, 29/114) to be used which increases the type I error within the trial. Finally, 90% power was used in 50% of the trials (57/114) with the less stringent 80% power used in 24% (27/114) of trials.

### Added benefit (superiority) on other outcomes

A range of added benefits were described within the review (Table 6), the most common being safety (28%, 32/114), typically represented by adverse events associated with the treatment. Patient-reported outcomes, convenience, cost and clinical outcomes were all also mentioned as the benefit of testing the new treatment.

**Table 6:** Added benefit described (superiority outcome)

Characteristic		Frequency (%) *N* = 114
**What is the main benefit of the new treatment?**	Safety	32 (28%)
	Patient-reported outcome measure	24 (21%)
	Convenience	18 (16%)
	Cost	18 (16%)
	Clinical outcome	16 (14%)
	None^a^	1 (1%)
	Unknown (not enough information)	5 (4%)
**Who is the main benefit for?**	Patient	70 (61%)
	Health service	28 (25%)
	Public health	11 (10%)
	None^a^	1 (1%)
	Unknown/unclear	4 (4%)

^a^One trial was designed as a NI trial when superiority was expected therefore no benefit was defined

Overwhelmingly, the main benefit of the new treatment was directly for the patient taking the treatment (61%, 70/114) for example with improvements in side effects or convenience of taking the treatment. However, in other cases, there was a benefit to the health service (25%, 28/114), e.g. reduced costs, or for public health, e.g. a reduction in antibiotic use across the population. All but one trial with enough information defined the added benefit expected by the treatment, however, 19 (/114, 18%) of all NI trials did not define a superiority outcome which would allow the evaluation of this added benefit within the trial.

Co-primary outcomes which included one NI outcome and one superiority outcome were used in just 18% (21/114) of the trials. A further 14/114 (12%) trials considered the statistical power for the superiority outcome even though they were not co-primary outcomes. Within the justification for the chosen NIM, the mention of the added benefit was present in 23% of cases (14/62). Many of these cases state the benefit in a general, unspecified manner, but some have quantified both the NI and superiority required for the new treatment to be deemed acceptable.

## Discussion

Following guidance and previous reviews that focus on regulatory NI trials, this review assessed the design characteristics of 114 publicly funded NI trials and evaluated their alignment to the recommendations for designing and analysing NI trials.

The median NIM was 8% for absolute risk differences (20% relative difference) and 0.35 as a standardised mean difference (SMD) for continuous outcomes, which is deemed to be a small to medium effect size [35]. Rothwell et al. [36] when evaluating superiority trials funded by NIHR’s Health Technology Assessment found a target effect size of 20% risk absolute difference and 0.35 SMD, these values would be expected to be larger than those for the NIM which is true for the absolute risk differences but the same value is being used when considering the SMD. A recent evaluation of cardiovascular trials with a mortality primary outcome found the median NIM chosen was higher than the median important difference within a superiority trial [37]. Additionally, the prevalent use of an absolute measure for risk differences ignores some of the statistical benefits when using a relative measure. NI trials that use an absolute measure are at risk of issues if there is a difference between expected and observed control event rate which can influence the appropriateness of the pre-defined NIM [38] and in the previously mentioned review of cardiovascular trials, 75% (18/24) of NI trials had a lower than expected control rate [37].

A stark finding of the review is the lack of reporting of the assumed difference between the treatment groups. This is an important element of the sample size calculation for non-inferiority trials [39], however, in 47% of cases, this information was omitted from the reporting making the calculation unreproducible. This is currently not mentioned within the CONSORT extension for reporting NI trials, however, the authors believe this should be an essential criterion when reporting the design of the trial.

Considering the justifications for the chosen NIM, there was a lower level of statistical considerations but higher levels of clinical considerations than in other reviews of NI trials. This may be due to the differing requirements for publicly funded trials, however, the lack of statistical considerations is especially alarming as the confirmation that the new treatment would be superior to placebo in an indirect comparison has not been confirmed. The increased level of confirming the value is clinically unimportant is encouraging, particularly with the regular inclusion of patient perspectives. This may be due to public funding streams where the inclusion of the patient voice is deemed essential and strongly encouraged [40]. Finally, against guidelines which suggest the using NIMs from other trials is not a suitable justification [41], the review found a fifth of NI trials used this as a rationale.

Most formal recommendations state that in NI trials, both ITT and PP populations should be used as the primary analysis populations [42]. Despite this, most of the trials selected to use the ITT population only, which could bias the results. Traditionally, it was expected that the ITT population would bias the results towards NI [43], due to the treatment effect being diluted by including non-compliant participants. However, a recent review into NI antibiotic trials suggested ITT may sometimes be the more conservative which the authors state may be due to lower success rates and larger variance in the ITT analysis [44]. Given this uncertainty, research suggests using additional analysis populations (inverse-probability-of-treatment weightings and doubly-robust estimators) to supplement the ITT and PP analyses [45] to enhance the robustness of results. One key consideration should be the estimand of interest to inform the most appropriate research question for the NI trials and help interpret if there are any differences between the results of analysis populations [43].

The benefit of the treatment is an important aspect of a NI trial, this benefit was clearly defined in almost all cases, but a related superiority outcome was not always included to assess this benefit within the trial. The superiority outcome was only statistically powered for in 30% of cases, most commonly as a co-primary outcome. By not powering for the superiority outcome, it implies that the success of the trial (and therefore the evaluation of the new treatment) is not dependent on a secondary outcome being declared superior as well as the primary NI outcome being declared non-inferior. Although this will be true for some treatments which are deemed to be more convenient for the patient, benefits such as improved safety (the most commonly used justification for completing a non-inferiority trial) should be confirmed within the trial to demonstrate the new treatment is a worthwhile alternative for use in practice.

A limitation of this review is the specific use of term “non-inferiority” which may not have been widely used before the 2012 guidelines and may partly be behind the large increase in NI trials found after this time. However, other potential terms such as “as good as” are very generic as a search term and so were not chosen for inclusion. The databases searched were chosen to maximise the potential to identify UK publicly funded NI trials, however, it is likely we have missed some NI trials, especially prone to being missed are non-NIHR funded trials which are being conducted abroad and so may favour a different registration database (i.e. ClinicalTrials.gov). Additionally, there were sometimes issues with access to relevant materials so many trials ended up having information categorised as unknown due to not enough information which may create a bias for those who have had their information made available.

## Conclusions

Publicly funded NI trials are increasing in popularity and therefore ensuring the guidelines (created with a focus on industry NI trials) are being suitably followed is important to ensure best practice. The NI trials in this review had high levels of reporting of the NIM with over half justifying this chosen value; however, the justification was more commonly based on clinical importance and statistical considerations (i.e. confirming superiority over placebo) were often absent. Researchers in the future should ensure this has been confirmed when designing NI trials to avoid a potential overlap with placebo effects. Additionally, despite recommendations to use both the ITT and PP population, the ITT population alone was used in most cases which is deemed to be the less conservative in terms of NI trials. This again, should be considered by researchers to ensure the NI trial is suitably powered for both important analysis populations.

## Data Availability

The dataset supporting the conclusions of this article is available in the University of Sheffield’s repository, https://doi.org/10.15131/shef.data.23623086.v1.

## Appendix

**Table:** Extraction spreadsheet summary for review of publicly funded NI trials

Section	Information extracted
Administration	Review Unique ID
	ISRCTN ID
	NIHR Award ID
	Title
	Institution
	Chief Investigator
	Funding amount
	Funder details (funder name, sub-committee, and call type as applicable)
	Documents used in the review and were thus deemed as sufficient information?
Trial characteristics	Trial start date
	Trial end date
	Project status
	Health category
	Number of treatment groups
	Treatment type (of each treatment in the trial)
	Randomisation type
	Primary analysis population/s
	Have co-primary outcomes been used?
	Primary non-inferiority outcome (including the category and type of outcome)
	Target sample size
Non-inferiority margin characteristics	Non-inferiority margin type (e.g. risk difference)
	Non-inferiority margin value
	Is the non-inferiority margin justified?
	Does the justification include a reference to clinical unimportant differences? (split further into clinician judgement, patient judgement and previously defined important differences)
	Does the justification include reference to statistical considerations?
	Does the justification include reference to previous non-inferiority trials?
	Does the justification include reference to published guidelines?
Statistical properties	Assumed difference between the two treatments
	Power (1-beta) used in the sample size calculation
	Significance level (alpha) used in the sample size calculation
	Standard deviation used in the sample size calculation (as applicable)
	Control rate used in the sample size calculation (as applicable)
Main benefit characteristics	Main benefit categorised
	Who is the main benefit for?
	Is the secondary outcome that represents the main benefit powered for?
	Is the added benefit mentioned in the justification for the non-inferiority margin?

## Abbreviations

CONSORT: Consolidated standards of reporting trials
IQR: Interquartile range
ISRCTN: International standard randomised controlled trial number
ITT: Intention-to-treat
NHS: National health service
NIHR: National institute for health research
NI: Non-inferiority
NIM: Non-inferiority margin
PP: Per protocol
SD: Standard deviation
SMD: Standardised mean difference
